# Dermatological Manifestations in Inflammatory Bowel Diseases

**DOI:** 10.3390/jcm10020364

**Published:** 2021-01-19

**Authors:** Elisabetta Antonelli, Gabrio Bassotti, Marta Tramontana, Katharina Hansel, Luca Stingeni, Sandro Ardizzone, Giovanni Genovese, Angelo Valerio Marzano, Giovanni Maconi

**Affiliations:** 1 Gastroenterology Section, Perugia General Hospital, 06156 Perugia, Italy; antelibetta@yahoo.com; 2 Gastroenterology & Hepatology Section, Department of Medicine, University of Perugia, 06156 Perugia, Italy; 3 Dermatology Section, Department of Medicine, University of Perugia, 06156 Perugia, Italy; marta.tramontana@unipg.it (M.T.); katharina.hansel@unipg.it (K.H.); luca.stingeni@unipg.it (L.S.); 4 Gastroenterology Unit, Department of Biomedical and Clinical Sciences, “L.Sacco” Hospital, 20157 Milano, Italy; sandro.ardizzone1@unimi.it (S.A.); giovanni.maconi@unimi.it (G.M.); 5 Dermatology Unit, Fondazione IRCSS Cà Granda, Ospedale Maggiore Policlinico, 20122 Milano, Italy; giovanni.genovese@unimi.it (G.G.); angelo.marzano@unimi.it (A.V.M.); 6 Department of Pathophysiology and Transplantation, University of Milano, 20122 Milano, Italy

**Keywords:** Crohn’s disease, dermatological manifestations, inflammatory bowel disease, skin, ulcerative colitis

## Abstract

Inflammatory bowel diseases (IBDs) may be associated with extra-intestinal manifestations. Among these, mucocutaneous manifestations are relatively frequent, often difficult to diagnose and treat, and may complicate the course of the underlying disease. In the present review, a summary of the most relevant literature on the dermatologic manifestations occurring in patients with inflammatory bowel diseases has been reviewed. The following dermatological manifestations associated with IBDs have been identified: (i) specific manifestations with the same histological features of the underlying IBD (occurring only in Crohn’s disease); (ii) cutaneous disorders associated with IBDs (such as aphthous stomatitis, erythema nodosum, psoriasis, epidermolysis bullosa acquisita); (iii) reactive mucocutaneous manifestations of IBDs (such as pyoderma gangrenosum, Sweet’s syndrome, bowel-associated dermatosis-arthritis syndrome, aseptic abscess ulcers, pyodermatitis–pyostomatitis vegetans, etc.); (iv) mucocutaneous conditions secondary to treatment (including injection site reactions, infusion reactions, paradoxical reactions, eczematous and psoriasis-like reactions, cutaneous infections, and cutaneous malignancies); (v) manifestations due to nutritional malabsorption (such as stomatitis, glossitis, angular cheilitis, pellagra, scurvy, purpura, acrodermatitis enteropathica, phrynoderma, seborrheic-type dermatitis, hair and nail abnormalities). An accurate dermatological examination is essential in all IBD patients, especially in candidates to biologic therapies, in whom drug-induced cutaneous reactions may assume marked clinical relevance.

## 1. Introduction

Inflammatory bowel diseases (IBDs) are a group of disorders characterized by chronic relapsing intestinal inflammation that includes two main entities—Crohn’s disease (CD) and ulcerative colitis (UC). Although, in the majority of cases, their signs and symptoms mainly involve the gastrointestinal tract, extra-intestinal manifestations (EIM) are relatively frequent [1]. Up to 40% of IBD cases may be complicated by EIMs and in some large series of studies, the prevalence of EIMs is higher in CD compared to UC [2]. Of note, almost every organ system may be affected in IBDs, including the eyes, skin, lungs, kidneys, and the hepatobiliary, immunologic, hematologic, and cardiovascular systems [2,3,4,5,6,7]. The skin is one of the most commonly involved organs, and cutaneous manifestations may be present in more than 10% of these patients [2,8], although higher rates have been documented [9,10].

The most common mucocutaneous lesions associated with IBDs are erythema nodosum (EN), pyoderma gangrenosum (PG), and aphthous stomatitis [11]. A prospective study conducted by Yüksel et al. in 352 IBD patients in a 4.5-year period concluded that cutaneous manifestations had a prevalence of 9.3%. The prevalence of EN (26 patients) and PG (8 patients) in IBDs was 7.4% and 2.3%, respectively [12].

The main goal of this review is to summarize the most recent knowledge regarding mucocutaneous manifestations related to IBD, focusing on secondary cutaneous manifestations due to concomitant drugs used for the treatment of these conditions.

## 2. Experimental Section

A comprehensive search of the electronic databases Medline and the Science Citation Index was made using the keywords “inflammatory bowel diseases”, “Crohn’s disease”, “ulcerative colitis”, “cutaneous manifestations”, “dermatologic manifestations”, “mucocutaneous manifestations”, “skin diseases”, and “extra-intestinal manifestations”, in various combinations with the Boolean operators *and*, *or*, and *not*. Only articles related to human studies were included, and manual cross-referencing was performed. Articles published in English between January 1970 and October 2020 were selected, but a search in languages other than English and books was also performed in our universities and other libraries.

## 3. Classification

**Figure 1:**
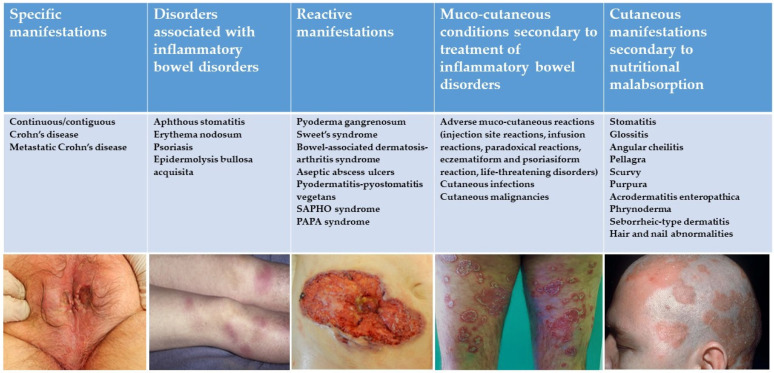
Mucocutaneous manifestations associated with inflammatory bowel disease (IBD).

Five categories have been identified to classify mucocutaneous manifestations associated with IBD based on their pathophysiological association with underlying disease (Figure 1), with their diagnostic approach, and specific and general treatments (Table 1):

1. Specific manifestations with the same histological features of the underlying IBD;
2. Mucocutaneous disorders associated with IBDs;
3. Reactive manifestations of IBDs due to immunological mechanisms triggered by common antigens shared by gut bacteria and skin;
4. Mucocutaneous conditions secondary to the treatment of IBDs;
5. Manifestations due to nutritional malabsorption.

### 3.1. Specific Manifestations with the Same Histological Features of the Underlying Inflammatory Bowel Disease

**Figure 2:**
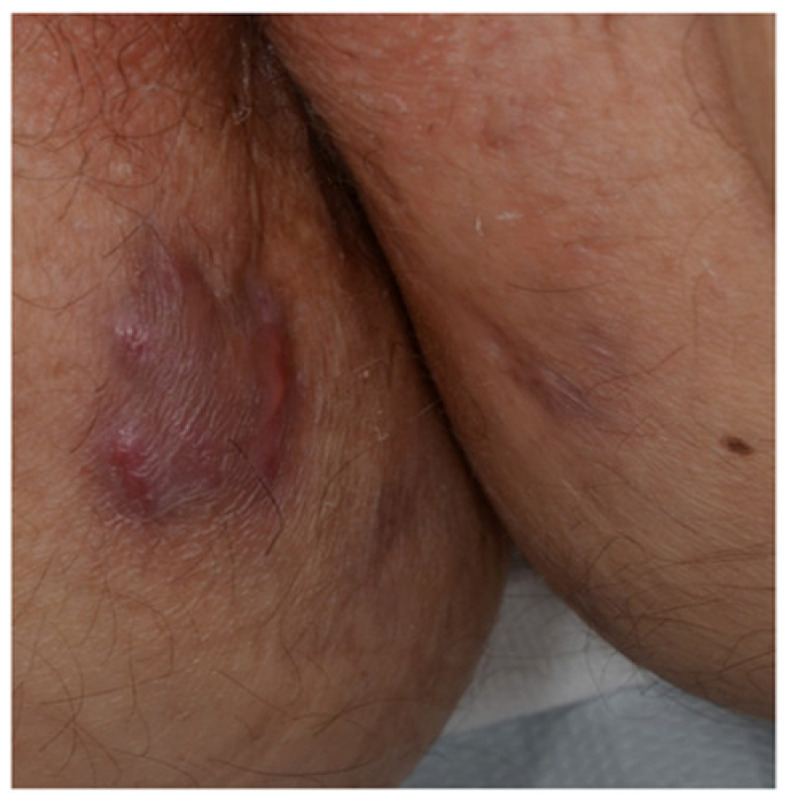
Erythematous plaque on the inner aspect of the right thigh of a patient with cutaneous Crohn’s disease.

These manifestations are the result of the intestinal inflammatory process diffusion into the skin and/or external mucosa. They are represented by “continuous/contiguous lesions” (Figure 2), including perianal/peristomal ulcers and orofacial lesions, and metastatic lesions, defined as non-caseating granulomas in sites distant from the gastrointestinal tract. The latter ones occur only in patients with CD, since UC lesions do not extend to external mucosal surfaces, and include fistula, abscesses, fissures, and ulcers.

#### 3.1.1. Continuous/Contiguous Lesions

Perianal disease occurs in about 50% of CD patients, and its severity ranges from perianal erythema to abscesses and perianal complex fistulae [13,14,15]. Due to the close proximity to the anus of perianal CD, perianal disease is considered as a continuous mucocutaneous manifestation, and not as an EIM in the European Crohn’s and Colitis Organization (ECCO) guidelines [1]. Entero-cutaneous fistulas have also been described in the abdominal scars of laparotomy or at the umbilicus [16].

#### 3.1.2. Metastatic Crohn’s Disease

The term “metastatic Crohn’s disease” (MCD) was coined by Mountain in 1970 [17]. However, some authors consider the term “noncontiguous cutaneous CD” more appropriate [18]. MCD is defined as the occurrence of specific non-caseating granulomatous cutaneous lesions at distant sites from the gastrointestinal tract. Although every body site can be affected, the most common involved areas are lower limbs and intertriginous areas [18]. The clinical manifestations of MCD consist of erythematous plaques, nodules, abscesses, fistulas, and ulcers. Sometimes, MCD may clinically resemble other conditions, such as erysipelas, cellulitis, or hidradenitis suppurativa [19,20,21]. No clear correlation between MCD development and the severity of underlying CD has been demonstrated. However, MCD tends to occur more frequently in patients with colonic or rectal involvement [21]. Since the polymorphic clinical presentation of MCD frequently leads to misdiagnoses, a detailed history and physical examination may provide crucial diagnostic clues. However, cutaneous histopathological assessment is mandatory for a definitive diagnosis [22].

### 3.2. Mucocutaneous Disorders Associated with IBD

Even though these disorders are relatively frequent in IBD patients, they may also occur in otherwise healthy subjects, and may sometimes be the first sign of intestinal disease. Their pathomechanisms are linked to the chronic inflammatory state and the expression of special human leukocyte antigen (HLA) genes such as HLA-DR2 and HLA-B27 [21]. They include aphthous stomatitis and erythema nodosum and, less frequently, psoriasis and epidermolysis bullosa acquisita.

#### 3.2.1. Aphthous Stomatitis

**Figure 3:**
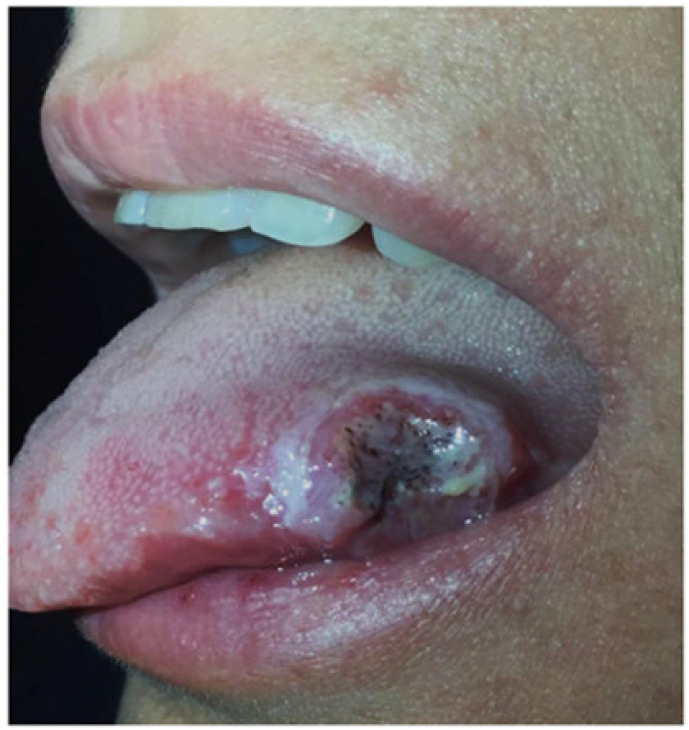
Aphthous stomatitis in a patient with Crohn’s disease.

Aphthous stomatitis is observed in about 10% of patients and is shared by UC and CD [23]. It is clinically characterized by multiple round or oval painful ulcers with a yellow pseudomembranous base and erythematous borders [24]. These ulcers are usually located in the buccal or labial mucosa (Figure 3), and their typical aspect makes histopathological evaluation usually unnecessary in patients with known IBD, whilst biopsy of borders and culture may be useful in persistent, recurrent, and refractory lesions, and in patients without a firm diagnosis of IBD [25].

Oral lesions are generally associated with active intestinal disease and HLA -B27 [26]. Control of IBD activity should always be attempted first and may lead to remissions of aphthous stomatitis. Topical treatment options are antiseptic mouthwashes and local steroids. Antitumor necrosis factor (TNF)-α agents may be effectively used in severe forms [27].

#### 3.2.2. Erythema Nodosum

**Figure 4:**
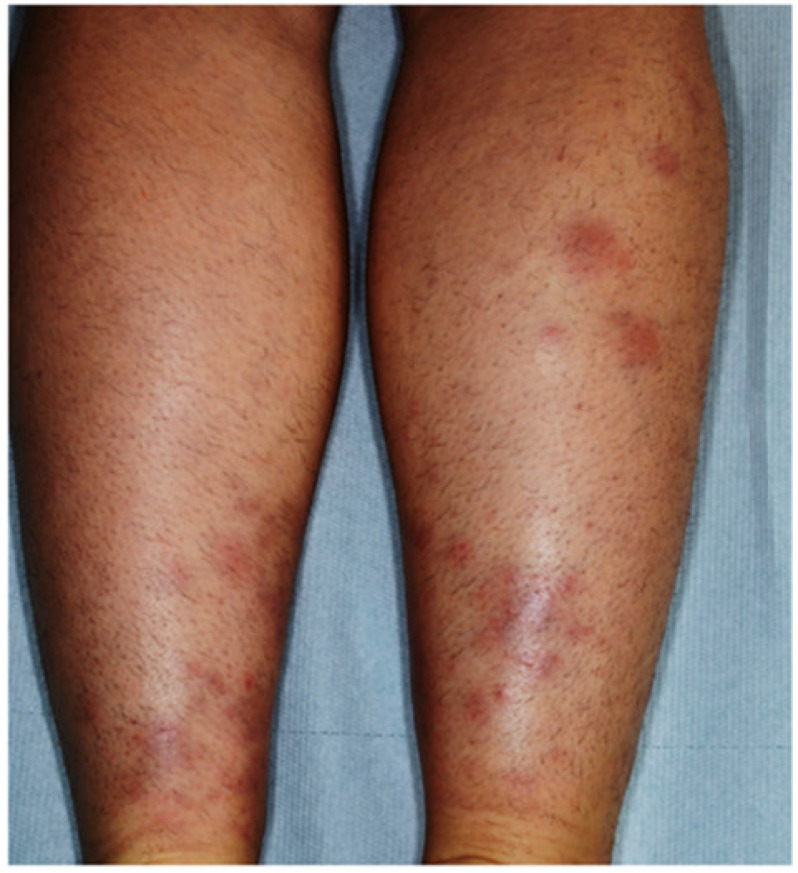
Erythematous nodules on the calves of a patient with ulcerative colitis-related erythema nodosum.

Erythema nodosum (EN) is the most common cutaneous manifestation of IBDs, affecting about 3–10% of UC patients and 4–15% of CD patients [1,28,29]. It appears more frequently in women [26], usually in concomitance with arthritis and active disease, and is positively affected by proctocolectomy. It is more common in IBD patients with HLA-B27 [26]. The time between UC diagnosis and EN onset is about 5 years [30] and EN in CD patients is mostly associated with colonic involvement [31]. EN is an acute inflammatory skin disease characterized by sudden onset of symmetrical, erythematous, warm, painful, and non-ulcerative nodules, ranging from 1 to 5 cm in diameter. They are mainly located on the extensor surfaces of the lower limbs (Figure 4), although they may involve any body site [32].

Face, trunk, and upper extremities are seldom affected. EN can simultaneously involve multiple locations and its recurrence rate is approximately 20% [33]. Of note, EN lesions correlate with the underlying disease activity and worsen with colitis flares in IBD patients [29], but not with the disease severity/extent. Systemic symptoms such as fever, malaise, and joint pain often occur. The typical course lasts for three to six weeks, but the residual bruise-like lesions can persist for months. Neither ulceration nor scarring occurs in EN. Skin biopsy is generally not necessary, as the diagnosis of EN is often based on the clinical presentation [34]. Nevertheless, the histological examination shows lympho-histiocytic infiltrate of the lower derma [11,13]. Most cases of EN are self-limited and do not require therapy, although bed rest and leg elevation are recommended to reduce the discomfort. In the case of pain, nonsteroidal anti-inflammatory drugs are considered as first-line treatment [35].

#### 3.2.3. Psoriasis

The link of psoriasis and IBD relies on three main epidemiologic links: (1) higher incidence of psoriasis in patients with Crohn’s disease (CD) and ulcerative colitis (UC), (2) predisposition to CD and UC in patients with psoriasis, and (3) high risk of iatrogenic psoriatic lesions in patients with IBD treated with anti-TNF agents. About 7–11% of patients with IBD develop psoriasis. Psoriasis is an erythematous-squamous disease occurring more frequently in CD (11.2%) than in UC (5.7%) [18]. The association between these conditions and the therapeutic overlap suggest shared inflammatory patterns and similar pathogenesis. There is no relationship between psoriasis severity and IBD activity [18]. These associations are partially explained by a common genetic background, as areas of chromosomes 16, 6, 4, and 3 share genetic markers of psoriasis and IBD. In particular, the IBD3 locus involved in CD and UC, and the PSORS1 locus involved in psoriasis, were found in the 6P21 region [36], and the gene encoding the interleukin 23 receptor (IL-23R) and interleukin 12B (IL-12B) are both implicated in the pathogenesis of psoriasis and IBD [37]. In addition, IBD and psoriasis share some common inflammatory pathways and are Th1-mediated inflammatory disorders associated with enhanced synthesis of cytokines (in particular, IL-17 and IL-21), TNF-α, and interferon-gamma (IFN-γ), which plays a relevant role in the pathogenesis of both conditions.

The iatrogenic link between IBD and psoriasis relies on several reports showing the development of psoriatic lesions in IBD patients treated with anti-TNF treatment, such as infliximab (IFX) or adalimumab (ADA). This paradoxical effect of anti-TNF treatment, which is effective in psoriatic patients, is not specific for IBD. It is observed in up to 2% of IBD patients treated with anti-TNF agents due to IBD [38], as well as other immune-mediated conditions, such as rheumatoid arthritis (RA) and ankylosing spondylitis.

The pathogenetic link between anti-TNF treatment in IBD and iatrogenic secondary psoriasis is still controversial, although it seems unlikely related to common genetic and pathogenic backgrounds (see below, Section 3.4.1) [39].

From a practical point of view, this association implies that IBD patients under anti-TNF agents have to be regularly examined for the potential development of secondary psoriatic lesions. The patients who develop psoriatic lesions can continue anti-TNF treatment, using topical treatment to control secondary psoriasis, unless the latter is not responding or is so severe, extending over 5% of body surface or complicating with pustules [40]. Anti-TNF treatment withdrawal may in fact result in exacerbation of the intestinal condition.

#### 3.2.4. Epidermolysis Bullosa Acquisita

About 30% of epidermolysis bullosa acquisita (EBA) patients are also affected by IBD, mostly CD [21]. EBA is an autoimmune mucocutaneous bullous disease caused by the autoantibodies against type VII collagen, which is associated with the MHC haplotype (HLA-DR2) [41]. Clinically, it is characterized by skin fragility, blister formation, and scarring, mainly localized at friction/clutch sites, such as hands, knees, and feet [42]. The co-occurrence of EBA and IBD seems to be pathogenically related to the “epitope-spreading phenomenon” [18]. Indeed, although specific proteins involved in this process have not been identified, it is hypothesized that chronic bowel inflammation in IBD patients may induce the development of autoantibodies against type VII collagen of the intestine. These autoantibodies would react with type VII collagen in the dermal–epidermal junction, inducing blister formation [43]. In IBD patients also affected by EBA, improvement of bowel disease induces skin lesions’ improvement [21].

### 3.3. Reactive Mucocutaneous Manifestation of IBD

Reactive mucocutaneous manifestations can be seen in UC and in CD. These conditions are characterized by histopathologically different findings compared to those observed in IBDs, but present a similar pathogenetic mechanism with abnormal innate immunity [21]. They include neutrophilic dermatoses, such as pyoderma gangrenosum (PG), pyodermatitis–pyostomatitis vegetans (PDPSV), Sweet’s syndrome (SS), bowel-associated dermatosis-arthritis syndrome (BADAS), and aseptic abscess syndrome. More rarely, SAPHO (synovitis, acne, pustulosis, hyperostosis, osteitis) syndrome and PAPA (pyogenic arthritis, pyoderma gangrenosum, acne) syndrome can also be associated with IBD as reactive conditions [18,21].

#### 3.3.1. Pyoderma Gangrenosum

**Figure 5:**
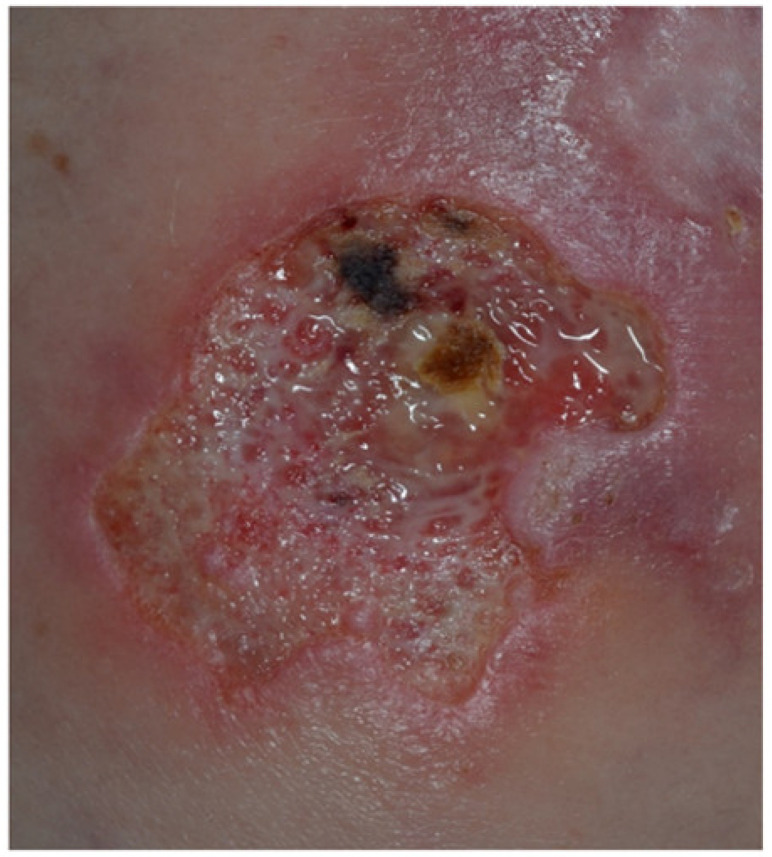
Ulcerative lesion with irregular violaceous, undermined borders on the right thigh of a patient with ulcerative colitis-associated pyoderma gangrenosum.

Pyoderma gangrenosum (PG) is a severe and often debilitating cutaneous IBD manifestation, occurring in about 1–2% of IBD patients [44]. It can occur before, during, or after the IBD onset and can display a course independent from IBD. PG is more frequently associated with UC, showing a female preponderance, and affecting more often patients of Black African origin and those with a positive UC family history [44]. Intriguingly, 50% of patients with PG have underlying IBD [44]. In most cases, the IBD predates PG onset, with less than 15% of PG cases appearing before IBD [44]. Although the pathophysiology of PG is not completely understood, it is nowadays regarded as autoinflammatory in its origin with a dysfunction of the innate immunity being crucial and adaptive immunity playing a contributing role [45,46,47,48]. PG, together with Sweet’s syndrome, is classified among the neutrophilic diseases, which encompass a huge group of forms due to accumulation and activation of neutrophils in the skin and, albeit rarely, in the internal organs [49,50]. Legs and peristomal skin are the most commonly involved sites, even though every body region may be affected. Clinical variants recognized in the literature are: (i) ulcerative; (ii) bullous; (iii) pustular; (iv) vegetative; (v) drug-induced; (vi) post surgical; (vii) peristomal. Ulcerative and pustular types are the most common ones related to IBDs [21]. PG initially presents with papules, pustules, or nodules that rapidly tend to ulcerate, developing a painful ulceration with typical violaceous raised undermined edges [51,52] (Figure 5).

The bed of the ulcer is necrotic and may be affected by secondary infection. Lesions can be isolated or multiple. Systemic symptoms, such as fever, arthromyalgia, and malaise may be associated with it. Lesions are often preceded by trauma because of the so-called “pathergy phenomenon” [48,52]. PG has a tendency to recur following successful treatment in more than 25% of cases, often in the same place as the initial episode [44]. PG diagnosis requires the exclusion of other possible skin diseases (e.g., ecthyma, necrotizing vasculitis, necrobiosis lipoidica, arterial or venous insufficiency ulceration) [53]. It is important to exclude infections because these may preclude the use of systemic corticosteroids and other immunosuppressants or biologics [54]. Underlying systemic diseases must be identified and treated. Histopathological evaluation from biopsies taken from the border of the lesion is useful to confirm the diagnosis, typically showing neutrophilic infiltrate with peripheric lymphocytic accumulation [48].

#### 3.3.2. Sweet’s Syndrome

**Figure 6:**
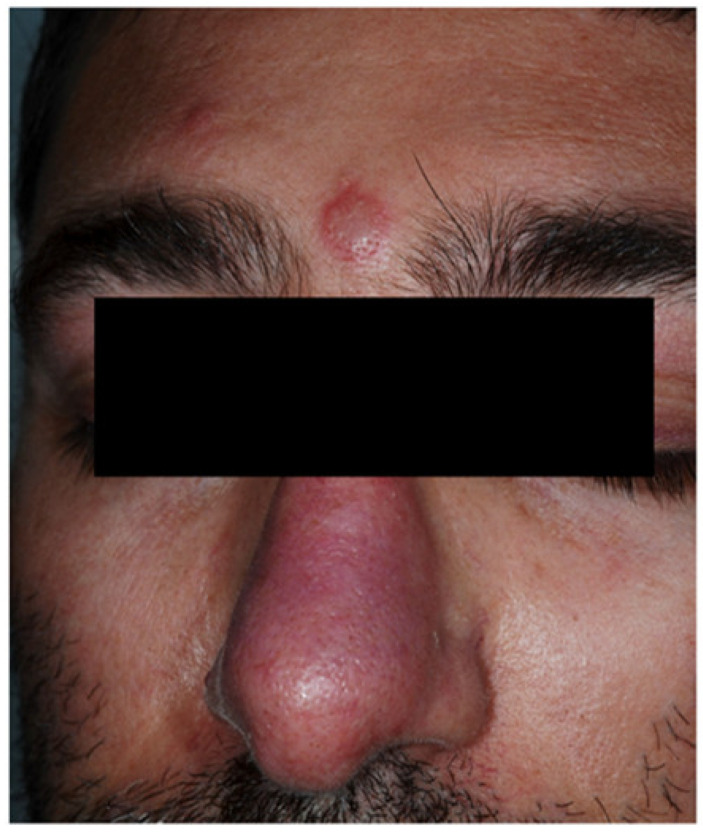
Erythematous papulonodular lesions involving the face of a patient with Sweet’s syndrome associated with Crohn’s disease.

Sweet’s syndrome is an acute neutrophilic dermatosis, firstly described by Sweet in the 1960s [18], and for the first time reported by Becuwe and collaborators as a possible disease associated with CD [55]. This association with IBD is not frequent and occurs more frequently in women than in men, mostly between the 3rd and 5th decade of life. Moreover, it seems more prevalent in CD than in UC, and in patients with colonic disease [18]. Sweet’s syndrome is characterized by erythematous papules and plaques, mainly involving the face (Figure 6), neck, and upper limbs. During the disease course, vesicles and pustules can occur, sometimes evolving into target-like lesions [50,56].

In most patients, cutaneous lesions are associated with systemic symptoms and signs, such as fever, arthromyalgia, headache, conjunctivitis, and oral ulcers [57]. Mucosal ulcerations in the esophagus, duodenum, and rectum may be seen at endoscopy [57]. Peripheral leukocytosis with neutrophilia and increase in erythrocyte sedimentation rate are frequently reported. Cutaneous lesions tend to spontaneously heal from weeks to months, without scarring. Edema of the papillary dermis and a dense neutrophilic infiltrate in the dermis represent the most consistent histopathological findings [50].

#### 3.3.3. Bowel-Associated Dermatosis-Arthritis Syndrome

Bowel-associated dermatosis-arthritis syndrome (BADAS) is a rare neutrophilic dermatosis, mainly described in patients after by-pass surgery for obesity [58]. Association with IBD is also reported [59]. BADAS is characterized by fever, arthromyalgia, abdominal pain, and skin involvement. Cutaneous lesions are usually polymorphic, mimicking several dermatoses, such as PG, hidradenitis suppurativa, and panniculitis [57]. Although its exact pathogenesis is unclear, it has been suggested that it may be due to an overgrowth of intestinal bacteria leading to the deposition of immune complexes in the skin and synovium [21].

#### 3.3.4. Aseptic Abscess Syndrome

Aseptic abscess syndrome is a rare condition, first described by Andre et al. in 1995 [60]. An underlying IBD is documented in around two-thirds of cases. Clinically, it presents with fever, abdominal pain, and aseptic neutrophilic abscesses, mainly localized in the spleen, although every organ can be involved. Abscesses and PG-like or Sweet-like lesions are the most frequently observed cutaneous manifestations [18].

#### 3.3.5. Pyodermatitis–pyostomatitis Vegetans

**Figure 7:**
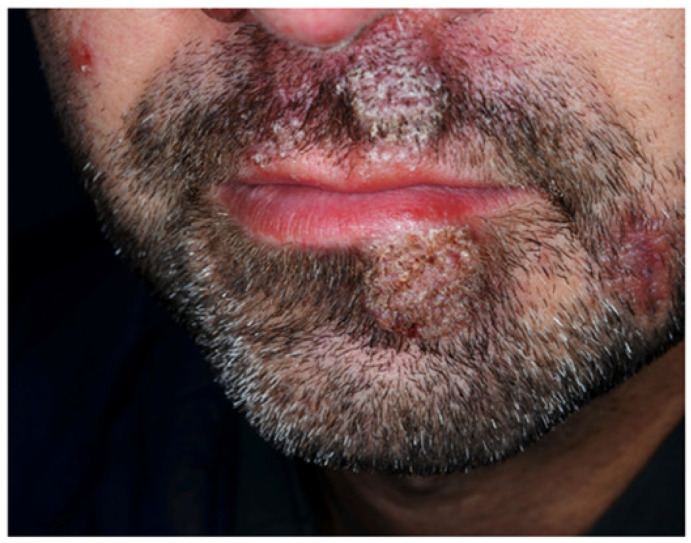
Vegetating plaques localized on the beard region of a patient with ulcerative colitis-associated pyodermatitis vegetans.

Pyodermatitis–pyostomatitis vegetans (PDPSV) is a rare mucocutaneous manifestation associated with IBD, more frequently with UC. Males are affected more frequently than women, with a M:F ratio of 3:1 [21,61]. The term “pyodermatitis vegetans” indicates an entity characterized by vesicular and papulopustular lesions, mainly localized in the scalp, face, axillae, and groins (Figure 7).

“Pyostomatitis vegetans” is the mucosal counterpart of “pyodermatitis vegetans”, and mainly involves the oral cavity. The typical presentation includes multiple small pustules with a characteristic “snail track-appearance” [62,63]. Coexistence of skin and mucosal lesions is possible. In about 90% of cases, peripheral blood eosinophilia is present. The clinical course of PDPSV parallels the activity of the underlying IBD [21]. The pathogenesis of pyodermatitis–pyostomatitis vegetans is still largely unknown. Although some correlation with infectious conditions such as nocardia vinacea, staphylococcus aureus, and HIV have been shown, a stronger correlation with IBD and other autoimmune and rheumatic disorders strongly supports its immune pathogenesis [64].

#### 3.3.6. SAPHO and PAPA Syndromes

SAPHO and PAPA syndromes are reactive manifestations of IBDs [21]. SAPHO syndrome is a seronegative spondyloarthropathy characterized by synovitis, acne, pustulosis, hyperostosis, and osteitis [65]. Besides acne conglobata or acne fulminans, cutaneous manifestations include dissecting cellulitis of the scalp and hidradenitis suppurativa. It is often associated with IBDs, particularly in young patients [21]. “PAPA syndrome” is characterized by pyogenic arthritis, pyoderma gangrenosum, and acne [65]. It is a genetic disorder with an autosomal dominant pattern. As well as SAPHO syndrome, it is mostly associated with UC [66].

### 3.4. Mucocutaneous Conditions Secondary to IBD Treatment

Several drugs used to treat IBDs can induce mucocutaneous conditions. In particular, in the last few years, anti-TNF-α agents used in IBDs have been increasingly reported to induce mucocutaneous side effects. TNF-α-antagonist-induced skin lesions include adverse mucocutaneous reactions, infectious complications, and skin cancers [67,68,69] (Table 2).

#### 3.4.1. Adverse Mucocutaneous Reactions

**Figure 8:**
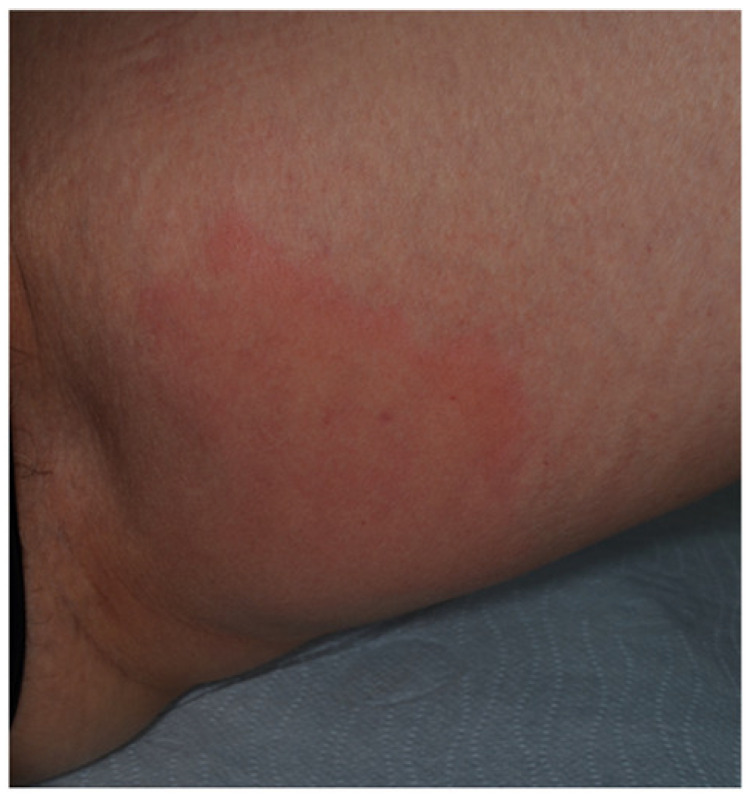
Erythematous-edematous reaction involving the injection site of etanercept in the left thigh.

The prevalence of cutaneous drug reactions induced by TNF-α antagonists among IBD patients ranges from 5 to 10% [67,70]. Anti-TNF-α agents can induce several skin adverse events, either localized or generalized (Table 2). Injection site reactions can occur following the administration of adalimumab, etanercept, and certolizumab pegol, being the most common cutaneous adverse events [71]. These events usually occur in the first month of treatment as erythematous-edematous, or eczematiform lesions in the injection site (Figure 8), associated with itch or pain, and typically disappear after 3–5 days, not requiring discontinuation of the drug. Infusion reactions can occur with infliximab and are distinguished into immediate and delayed hypersensitivity reactions.

**Figure 9:**
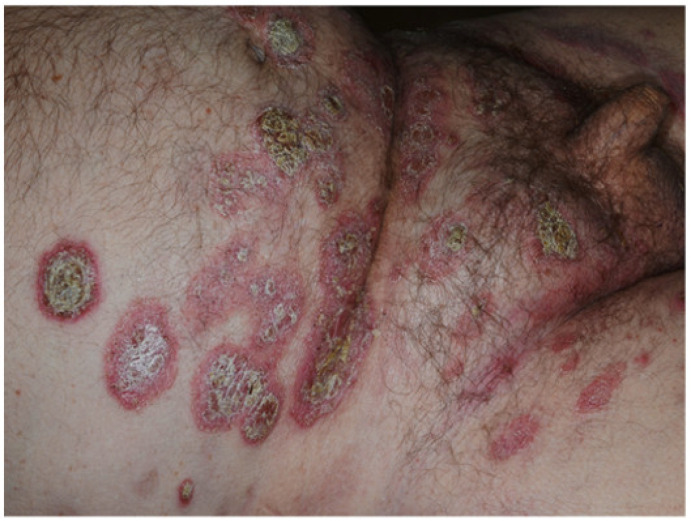
Psoriasiform eruption involving the abdomen and the pubic area of a patient undergoing adalimumab treatment.

The latter ones are the most frequently observed, presenting 1–2 weeks after the infusion as “serum sickness-like reactions” characterized by an erythematous rash, hand and facial edema, fever, and arthromyalgias. On the other hand, immediate hypersensitivity reactions occur in the first two hours after infusion starting and are characterized by the appearance of urticarial lesions diffuse erythematous eruption, and anaphylaxis [72]. TNF-α antagonists can cause “paradoxical” reactions, defined as new onset (80% of cases) or exacerbation (20% of cases) of preexisting dermatoses that usually are treated with this class of drugs [67,68,70]. These reactions encompass psoriasis (Figure 9), hidradenitis suppurativa, lupus erythematous, dermatomyositis, vasculitis, bullous dermatoses, granuloma annulare, lichen planus, PG, alopecia areata, vitiligo, and sarcoidosis [71].

The underlying pathogenic mechanisms of paradoxical reactions in IBDs is still poorly understood and several hypotheses have been proposed. Epidermal permeability barrier dysfunction, increased susceptibility to bacterial superinfection, interferon-γ-secreting Th1 cells, Th17 cells (interleukin-17A and IL-22), plasmacytoid dendritic cells (interferon-α), and keratinocytes (IL-36γ and IL-17C) have been suggested [73]. Female sex, CD, personal or familial history of inflammatory skin diseases, smoking, an increased body mass index, and treatment with adalimumab have been identified as independent predictive factors for developing paradoxical reactions [66,74]. In an observational study carried out in 85 IBDs patients, Rahier et al. [38] assessed the clinical characteristics, risk factors, and outcomes of psoriatic and eczematous lesions induced by anti-TNF-α in patients with IBD. The same authors found that 5% of patients included in their cohort developed anti-TNF-α-induced skin lesions during a follow-up period of 4 years. Skin lesions were more frequent among females and in patients with a positive family history for inflammatory skin disorders such as psoriasis or atopic dermatitis. In an observational, case–control Spanish study, the cumulative incidence of psoriasis was 1.0% after 1 year from starting anti-TNF-α treatment, 2.5% after 5 years, and 4.5% after 10 years [75]. Recently, a much higher incidence of psoriasis of up to 10.5% has been described in a retrospective analysis; in the same study, female gender, foregut disease location, and fistulizing and stricturing disease appeared to be risk factors for anti-TNFα-induced psoriasis. Interestingly, adalimumab therapy was found to induce earlier psoriasis lesions than infliximab [76].

**Figure 10:**
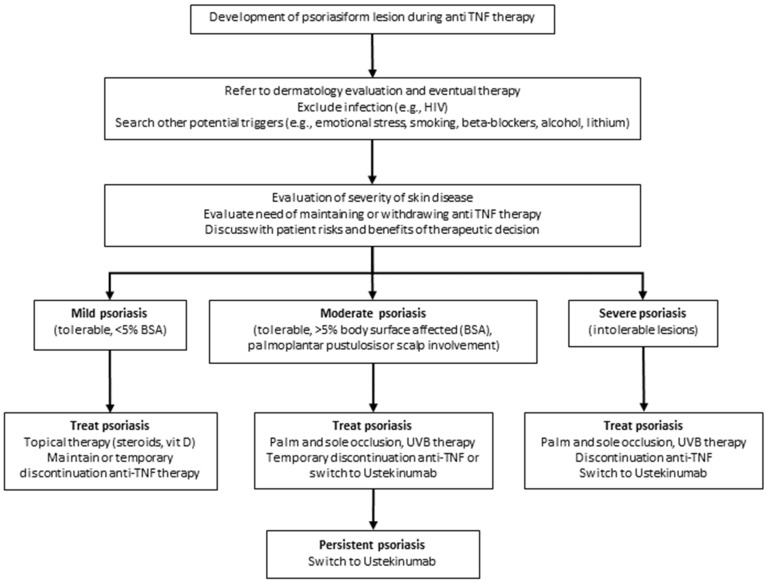
Proposed algorithm for psoriasis management in IBD.

Pustular psoriasis is reported in 56%, plaque psoriasis in 55%, and guttate psoriasis in 15% of patients treated with anti-TNF-α agents; pustular lesions occurred mainly on palms and/or soles [77]. In addition, eczematous and psoriasis-like skin eruptions have also been described [78]. In an IBD cohort of 1004 patients previously treated with anti-TNF-α agents including infliximab, adalimumab, and certolizumab, psoriasis-like lesions were observed in 27 patients, mostly affected by CD [79]. In this study, the mean time between appearance of psoriasiform lesions and the start of anti-TNF-α therapy was shorter in patients receiving adalimumab (40.7 weeks) than in those undergoing infliximab (126.9 weeks) or certolizumab (63.5 weeks). Management of psoriasiform lesions in IBD patients under anti-TNF agents includes topical therapy and if possible, anti-TNF temporary suspension or withdrawal, or switching with ustekinumab (Figure 10). Finally, potentially life-threatening disorders, such as urticaria-angioedema [80], anaphylaxis, Stevens–Johnson syndrome, and toxic epidermal necrolysis have also been reported [71].

#### 3.4.2. Infectious Complications

An increased prevalence of bacterial, viral, and fungal opportunistic infections in patients treated with anti-TNF-α agents has been reported. Skin infections induced by bacteria, i.e., erysipelas, cellulitis, and abscesses have been described in about 0.1–7% of patients [72]. Reactivation of herpes viruses, particularly of varicella zoster virus (VZV), was documented in about 3% of patients treated with TNF-antagonists, mostly infliximab and adalimumab [81,82]. ECCO consensus recommends to not start biologic therapy during an active infection and to discontinue treatment in the case of reactivation of herpes viruses [83].

Mucocutaneous viral infections induced by human papilloma virus, molluscum contagiosum virus, and cytomegalovirus have also been reported in psoriatic patients under anti-TNF-α agent treatment [72].

#### 3.4.3. Skin Cancers

The increased incidence of skin cancers in patients treated with anti-TNF-α agents is still controversial. Some authors reported an increased risk for nonmelanoma skin cancers, particularly basal cell carcinomas [71]. Cutaneous lymphomas such as mycosis fungoides and Sézary syndrome have been documented more frequently in patients treated with a combination of TNF-α -inhibitors and thiopurines [71].

### 3.5. Manifestations Due to Nutritional Malabsorption

In IBD patients, skin lesions can also be caused by nutritional deficiencies, such as stomatitis, glossitis, angular cheilitis (vitamin B), pellagra (niacin), acrodermatitis enteropathica (zinc), scurvy (vitamin C), and purpura (vitamin C and K). Rarely, phrynoderma, seborrheic-type dermatitis, and hair and nail abnormalities have also been reported [21].

## 4. Conclusions

Mucocutaneous manifestations in IBD patients are relatively frequent either in treated and untreated subjects, and may be of considerable clinical impact. For this reason, a dermatologic evaluation is of paramount importance in these patients, and should be mandatory in candidates for biologic therapies, especially anti-TNF-α agents. A strict collaboration between gastroenterologists and dermatologists in the assessment and management of IBD patients is desirable in all centers involved in the care of these subjects.

## Figures and Tables

**Table 1:** Diagnostic and therapeutic management of manifestations associated with IBD.

Manifestations	Diagnostic Approach	Specific Treatment	General Treatment in Relation to IBD Activity or IBD Treatment
**Specific manifestations**			
Continuous/contiguous Crohn’s disease	Refer to dermatologist/biopsy	Surgical approach	Control inflammation
Metastatic Crohn’s disease	Refer to dermatologist/biopsy	Surgical approach	Control inflammation
**Disorders associated with IBD**			
Aphthous stomatitis	Refer to dermatologist/stomatologist	Topical corticosteroids, colchicine	Control inflammation
Erythema nodosum	Refer to dermatologist/rule out other causes of erythema nodosum	Pain management (NSAIDs, colchicine), bed rest, systemic corticosteroids	Control inflammation
Psoriasis	Refer to dermatologist	Topical corticosteroids/vitamin D derivatives, systemic retinoids, methotrexate, cyclosporine, TNF inhibitors, IL-12/IL-23 inhibitor (ustekinumab), IL-17 inhibitors, IL-23 inhibitors, apremilast	Control inflammation
Epidermolysis bullosa acquisita	Refer to dermatologist/biopsy and perform direct and indirect immunofluorescence test and ELISA	Systemic steroids ± steroid-sparing immunomodulating agents (dapsone) or immunosuppressants (azathioprine, mycophenolate mofetil)	Control inflammation
**Reactive manifestations**			
Pyoderma gangrenosum	Refer to dermatologist/biopsy	Systemic corticosteroids, dapsone, cyclosporine, TNF inhibitors, IL-12/IL-23 inhibitor (ustekinumab), IL-1 receptor antagonist (anakinra), anti-IL-1β monoclonal antibody (canakinumab)	Control inflammation
Sweet’s syndrome	Refer to dermatologist/biopsy	Systemic corticosteroids	Control inflammation
Bowel-associated dermatosis-arthritis syndrome	Refer to dermatologist/rheumatologist	Systemic antibiotics, systemic corticosteroids	Control inflammation
Aseptic abscess ulcers	Refer to dermatologist/biopsy	Systemic corticosteroids	Control inflammation
Pyodermatitis–pyostomatitis vegetans	Refer to dermatologist/biopsy	Systemic steroids ± steroid-sparing agents, colchicine, systemic steroids, bisphosphonates, methotrexate, sulfasalazine, TNF inhibitors, IL-1 receptor antagonist (anakinra)	Control inflammation
SAPHO syndrome	Refer to dermatologist/rheumatologist	Systemic steroids, IL-1 receptor antagonist (anakinra)	Control inflammation
PAPA syndrome	Refer to dermatologist/rheumatologist/biopsy		Control inflammation
**Mucocutaneous conditions secondary to treatment of IBD**			
Adverse mucocutaneous reactions (injection site reactions, infusion reactions, paradoxical reactions, eczematiform and psoriasiform reaction, life-threatening disorders)	Refer to dermatologist	Different treatments according to the adverse mucocutaneous reaction	Consider to withdraw/change IBD treatment
Cutaneous infections	Refer to dermatologist	Systemic/topical antibiotics	Consider to withdraw/change IBD treatment
Cutaneous malignancies	Refer to dermatologist/biopsy	Surgical excision/topical treatment	Consider to withdraw/change IBD treatment
**Cutaneous manifestations secondary to nutritional malabsorption**			
Stomatitis	Refer to dermatologist	Start supplementation	Start supplementation
Glossitis	Refer to dermatologist	Start supplementation	Start supplementation
Angular cheilitis	Refer to dermatologist	Start supplementation	Start supplementation
Pellagra	Refer to dermatologist	Start supplementation	Start supplementation
Scurvy	Refer to dermatologist	Start supplementation	Start supplementation
Purpura	Refer to dermatologist	Start supplementation	Start supplementation
Acrodermatitis enteropathica	Refer to dermatologist	Start supplementation	Start supplementation
Phrynoderma	Refer to dermatologist	Start supplementation	Start supplementation
Seborrheic-type dermatitis	Refer to dermatologist	Start supplementation	Start supplementation
Hair and nail abnormalities	Refer to dermatologist	Start supplementation	Start supplementation

Abbreviations: NSAIDs—nonsteroidal anti-inflammatory drugs; TNF—tumor necrosis factor; ELISA—enzyme-linked immunosorbent assay.

**Table 2:** Mucocutaneous conditions secondary to anti-TNF agents.

**Adverse mucocutaneous reactions**
Injection site reactions
Infusion reactions
“Paradoxical” reactions
Eczematous and psoriasis-like reactions
Life-threatening disorders (urticaria-angioedema, anaphylaxis, Stevens Johnson syndrome, and toxic epidermal necrolysis)
**Cutaneous infections**
Bacterial (erysipelas, cellulitis, and abscesses)
Viral (herpes, cytomegalovirus, papillomavirus, etc.)
Fungal
Opportunistic infections
**Cutaneous malignancies**
Non-melanoma skin cancers (basal cell carcinomas, squamous cells carcinomas)
Cutaneous lymphomas (mycosis fungoides, Sézary syndrome)
